# Supplementary material for: Comparative Benchmarking of Optical Genome Mapping and Chromosomal Microarray Reveals High Technological Concordance in CNV Identification and Additional Structural Variant Refinement
Source: Genes (Basel). 2023 Sep 26;14(10):1868. doi: 10.3390/genes14101868 (PMC10667989; doi:10.3390/genes14101868)
Supplement: Supplementary file 1 [file genes-14-01868-s001.zip › Figure S1.pdf]

A.

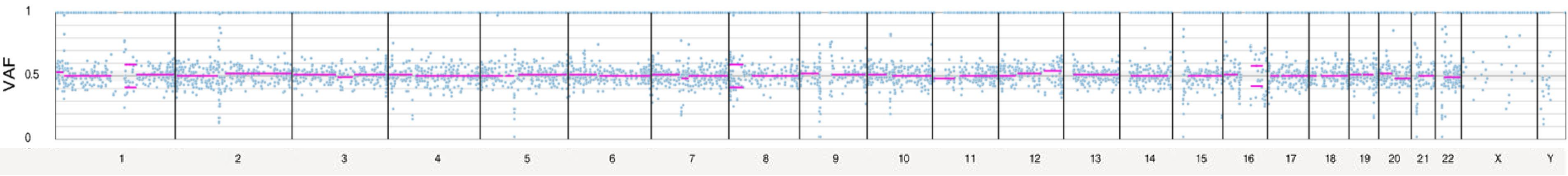

B.

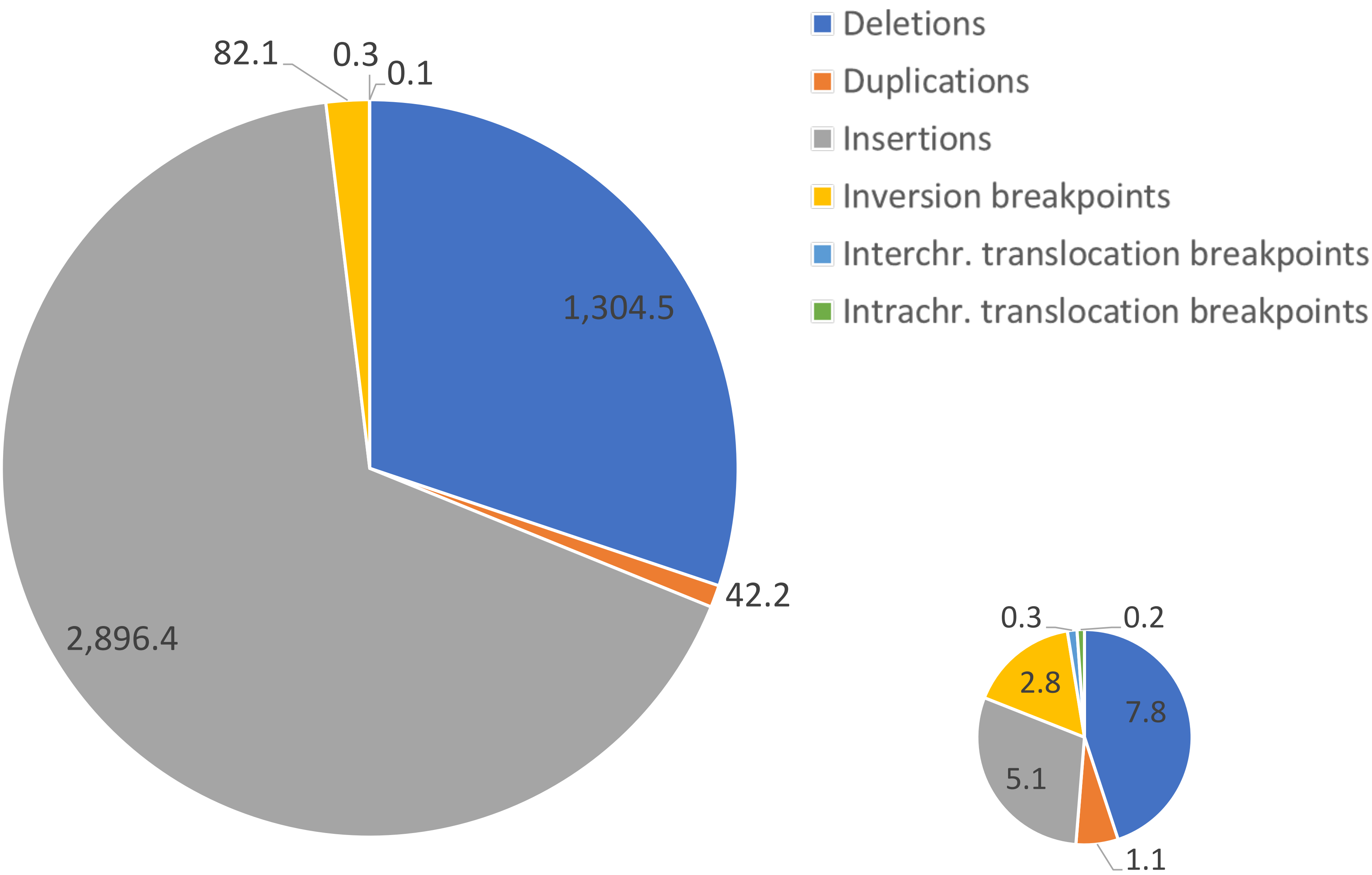

C.

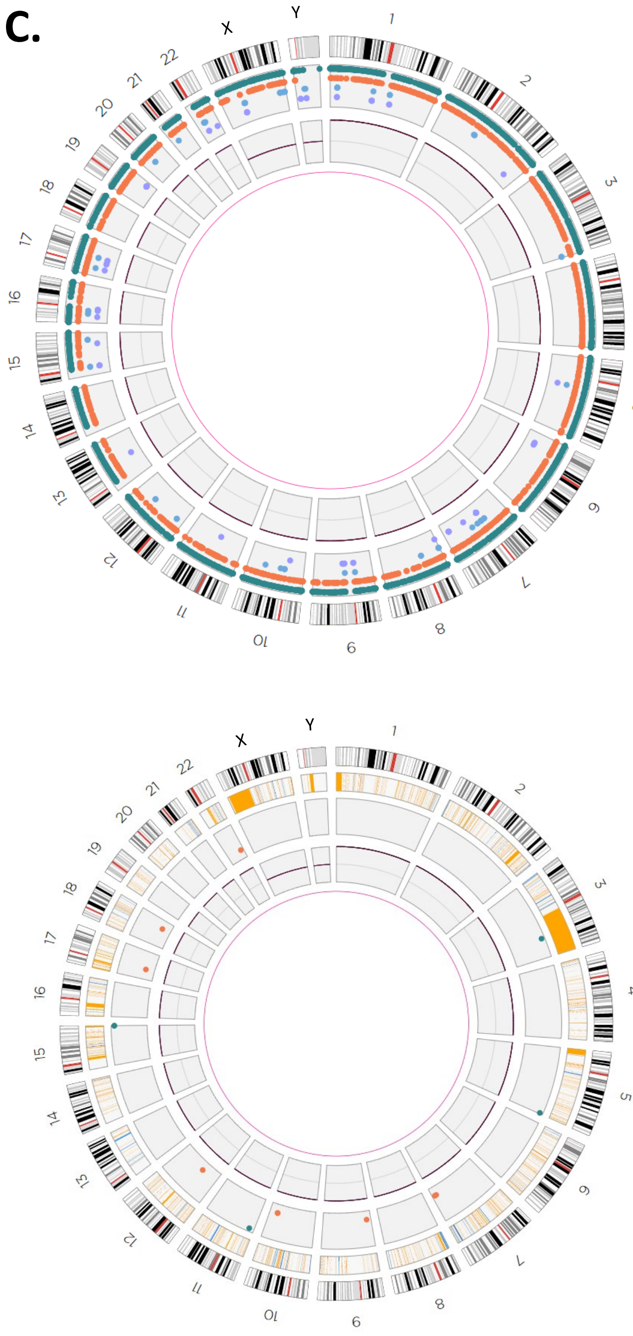

	All	Post filter
Deletions	1317	9
Duplications	42	0
Insertions	2889	5
Inversions	90	4
Interchr translocations	0	0
Intrachr translocations	0	0
**Total**	**4338**	**17**

Known regions associated with constitutional disorders
