# Supplementary figures and images for: Comparative Benchmarking of Optical Genome Mapping and Chromosomal Microarray Reveals High Technological Concordance in CNV Identification and Additional Structural Variant Refinement
Source: Genes (Basel). 2023 Sep 26;14(10):1868. doi: 10.3390/genes14101868 (PMC10667989; doi:10.3390/genes14101868)

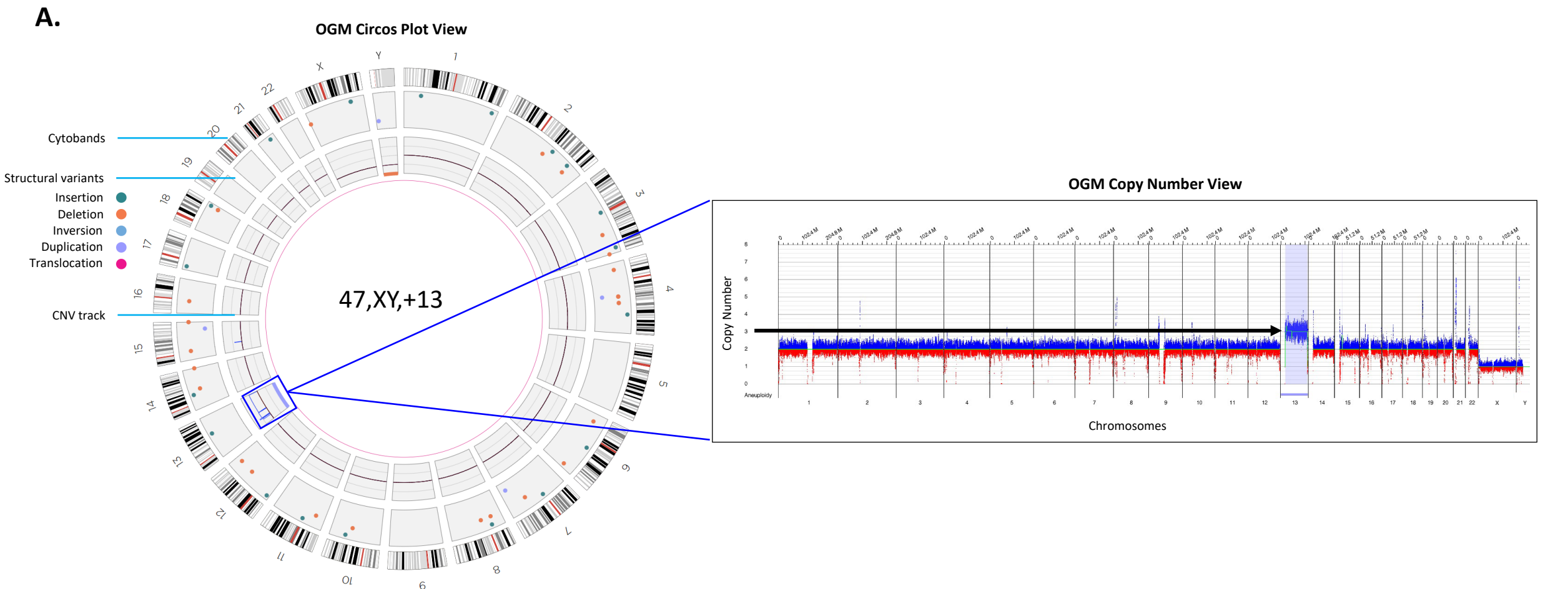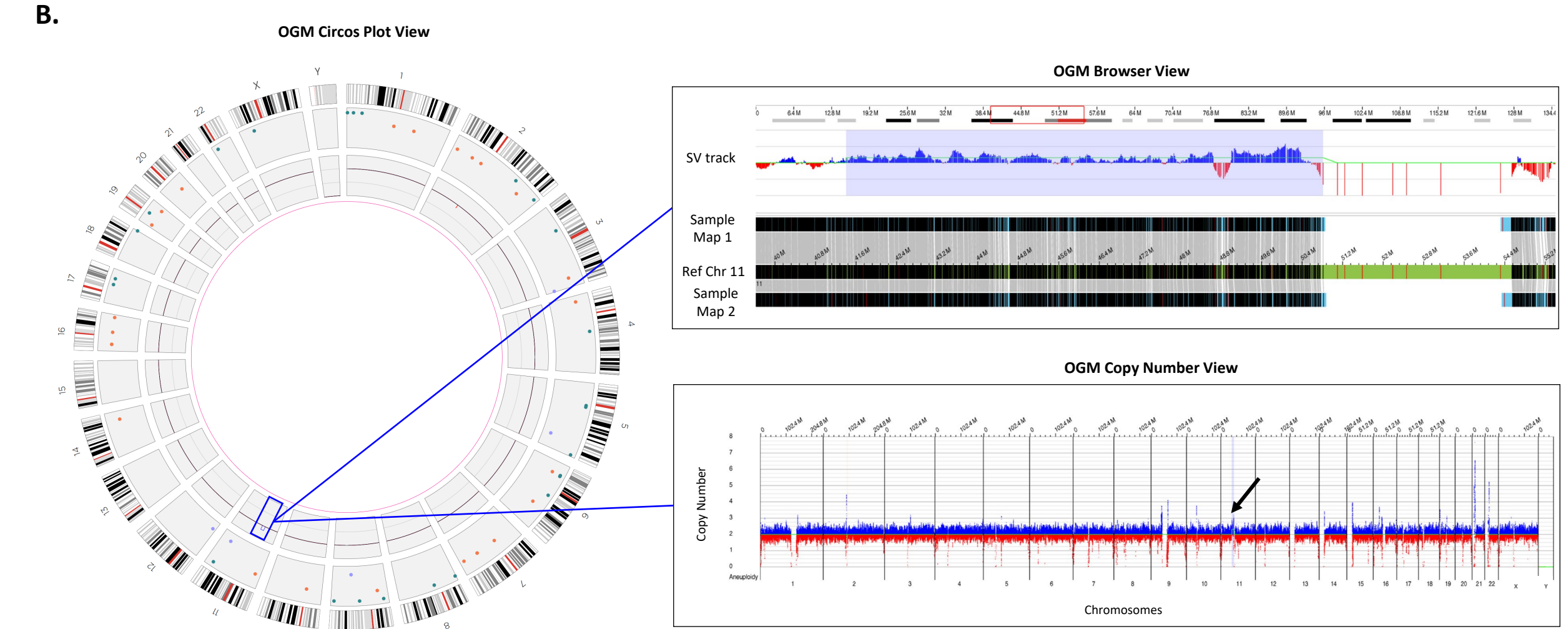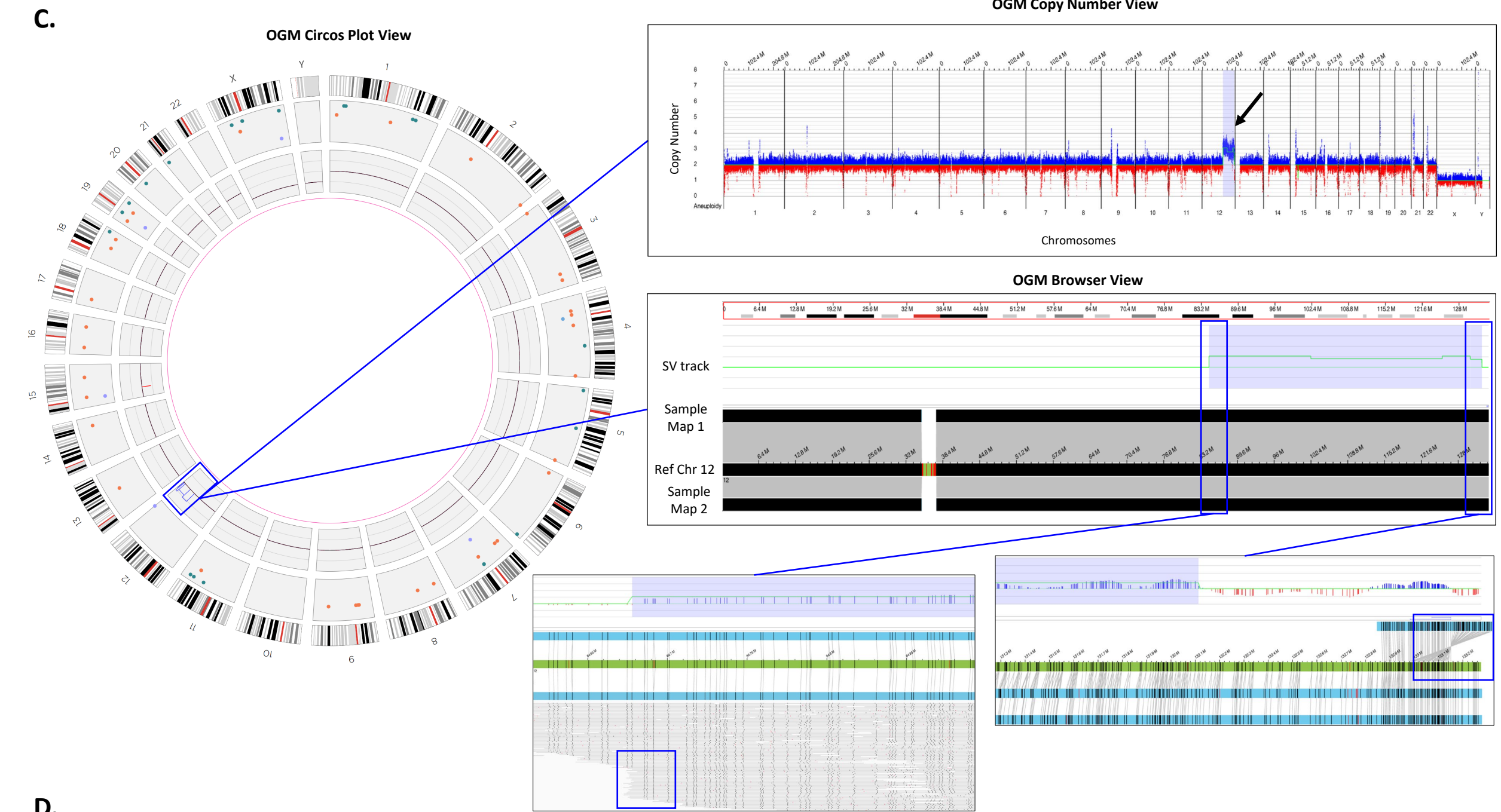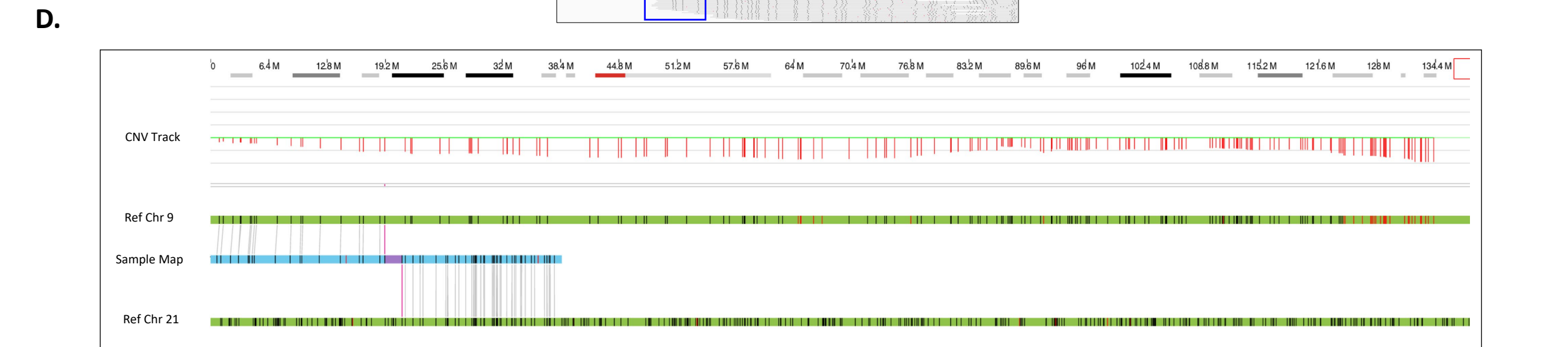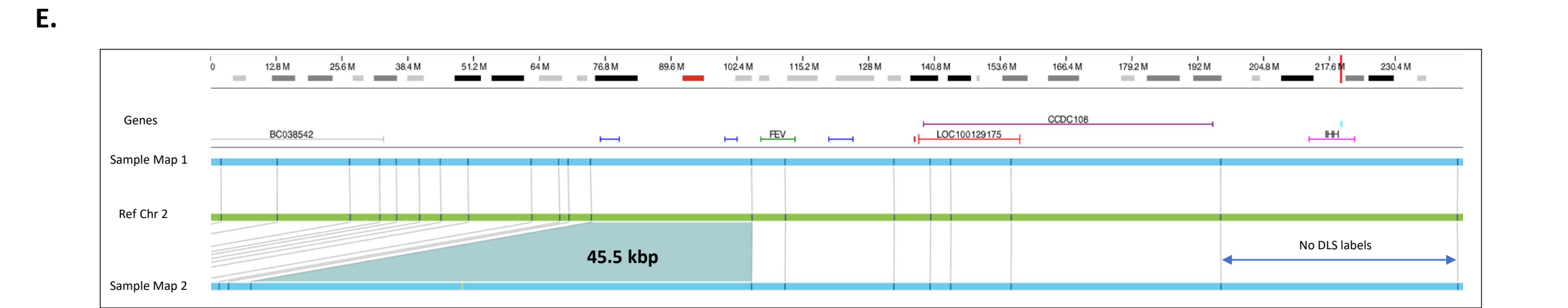

Supplement: Supplementary file 1 [file genes-14-01868-s001.zip › Figure S2.pdf]
